# Clinical study of functional neuronavigation combined with intraoperative fluorescein sodium guided technique in the surgery of brain metastases

**DOI:** 10.3389/fonc.2026.1942300

**Published:** 2026-09-28

**Authors:** Gai Hang, Weiran Yang, Zhong Wang

**Affiliations:** Department of Neurosurgery, Inner Mongolia People's Hospital, Hohhot, China

**Keywords:** brain metastases, guided technique, neuronavigation, sodium fluorescein, surgery

## Abstract

**Purpose:**

To evaluate the efficacy of functional neuronavigation combined with intraoperative fluorescein sodium in the operation of brain metastases.

**Patients and methods:**

From January 2018 to December 2023, 80 patients with brain metastases were divided into observation and control groups based on using sodium fluorescein during surgery. Magnetic resonance image (MRI) and other imaging sequences, were performed, and the image data were imported into Brainlab functional nervous navigation software before the operation. The specific location of the tumor tissue, cone bundle, the spatial relationship of the great vessels, and image fusion with the skull multi-plane computed tomography was used to determine the surgical navigation plan. A standardized sodium fluorescein allergy test was performed after induction of general anesthesia, with predefined diagnostic criteria for hypersensitivity. Intravenous low-dose fluorescein sodium (2 mg/kg) was administered following a negative test. Intraoperatively, neuronavigation was combined with sodium fluorescein to perform tumor resection by displaying the border between tumor tissue and normal brain tissue using the YELLOW 560 fluorescence mode of a Zeiss Pentero 900 microscope.

**Results:**

The observation group revealed a significantly better tumor resection outcome than the control group, with a statistically significant difference (*X*2 = 6.144, P = 0.013). Postoperative Karnofsky performance status scores in the observation group were also significantly improved than the control group (*X*2 = 4.286, P = 0.038) Additionally, the observed group experienced fewer new-onset functional impairments postoperatively, with a statistically significant difference (*X*2 = 4.800, P = 0.028). Although the observation group had fewer postoperative complications than the control group, this difference was not statistically significant (*X*2 = 2.294, P = 0.130). The surgical time in the observation group (216.65 ± 56.76) was shorter than the control group (225.62 ± 37.68), however, the difference was not statistically significant (t=0.589, P = 0.560). Similarly, the length of hospitalization in the observation group (21.48 ± 3.23) was lower than that in the control group (22.43 ± 2.34), however, the difference was statistically insignificant (t=1.065, P = 0.294).

**Conclusion:**

Multimodal functional neuronavigation combined with intraoperative sodium fluorescein guidance is a safe and feasible technique for brain metastasis surgery. It enables accurate tumor localization, delineates the boundary between the tumor and surrounding normal brain tissue, increases the extent of resection, and reduces postoperative neurological deficits without increasing complications, thereby improving patients’ quality of life and functional outcomes.

## 1 Introduction

Brain metastases (BMs) are among the most common malignant tumors in adults, accounting for 20% of all intracranial malignant. With the increase in the incidence of systemic tumors and improvements in diagnosis and treatment technologies, patients’ survival has improved, leading to an upward trend in the incidence of brain metastases (1, 2). Among all malignant, lung, breast, kidney, melanoma, and colorectal cancers are the most prone to brain metastasizing to the brain. Surgical resection still plays an important role in the treatment of brain metastases (3, 4). Accepted indications for craniotomy include large metastases (>3 cm in diameter); impending herniation from severe peritumoral edema or intratumoral hemorrhage; lesions suspected to be metastases without confirmed primary pathology; and cystic or radioresistant metastases. Conversely, small, deep-seated, asymptomatic lesions in non-eloquent regions are often preferentially managed with stereotactic radiosurgery (SRS) or whole-brain radiotherapy (WBRT) (11–13). However, safely achieving tumor resection without causing neurological dysfunction remains challenging (5). Neuronavigation combined with intraoperative fluorescein sodium guidance technology, has been widely used in surgeries for high-grade gliomas (6); However, reports on its integrated application in brain metastases resection remain limited. To our knowledge, the combination of low-dose sodium fluorescein with multimodal functional neuronavigation—incorporating diffusion tensor imaging (DTI), magnetic resonance angiography (MRA), and functional mapping—has not been systematically described in this context. This study retrospectively analyzed the application of navigation combined with fluorescein sodium guidance in the surgical resection of brain metastases, with the aim of providing valuable references for precise surgical treatment.

## 2 Materials and methods

### 2.1 General information

Eighty patients with brain metastases who underwent surgical treatment at the Department of Neurosurgery of the Inner Mongolia Autonomous Region People’s Hospital between January 2018 and December 2023 were selected for this study. All patients underwent functional neuronavigation-guided surgery. They were divided into observation and control groups according to whether fluorescein sodium was used during the operation for retrospective analysis. The observation group consisted of 40 patients (32 males and eight females) while the control group comprised 40 patients (26 males and 14 females). Disease duration ranged from 2 weeks to 3 months. Detailed information is presented in **Table 1**. Patients in the fluorescein-guided group (January 2020 – December 2023) and control group (January 2018 – December 2019) were enrolled consecutively based on surgery date; grouping was non-randomized. Baseline characteristics, including age, tumor size, and primary cancer type, showed no significant differences after propensity score matching (see **Table 1**), suggesting good comparability between groups. All procedures were performed by three neurosurgical teams. Because the combined fluorescein-navigation protocol was introduced by one senior team in January 2020, the distribution of teams was not uniform between groups: the control group (2018–2019) included cases from all three teams, whereas the observation group (2020–2023) was weighted toward the team that implemented the protocol. To mitigate potential surgeon-related confounding, surgeon experience (years in independent practice) was included as a covariate in the propensity score matching model.

**Table 1:** Comparison of general information between the control group and the observation group.

Clinical characteristics	Control group (n=40)	Observation group (n=40)	Statistical value	P value
Gender (male/female)	26/14	32/8	χ^2^=1.129	0.288
Age (years)			χ^2^=0.125	0.723
≥60	30	28		
<60	10	12		
Tumor location(%)			χ^2^ = 0.000	1.000
Supratentorial	34	32		
Infratentorial	6	8		
Preoperative KPS score			χ^2^ = 0.125	0.723
≥70	30	28		
<70	10	12		
Number of metastatic tumors			χ^2^ = 0.092	0.338
1	20	26		
2	14	12		
≥3	6	2		
Metastasis in other organs			χ^2^ = 0.574	0.449
Yes	12	6		
No	28	34		
Pathological type			χ^2^ = 0.114	0.736
Lung cancer	26	28		
Gastrointestinal tumor	4	6		
Genitourinary tumor	6	4		
Breast cancer	4	2		

Inclusion criteria are as follows:

(1) Patients with brain metastases who consented to surgery, were over 18 years old and had an initial diagnosis. (2) Patients with relatively large metastatic tumors (diameter greater than 3 cm). (3) Patients with manifestations of brain herniation due to severe peritumoral edema or tumor stroke. (4) Patients with intracranial space-occupying lesions suspected to be brain metastases and with unclear pathology, as well as cystic brain metastases that are not sensitive to radiotherapy, chemotherapy, or targeted therapy. (5) No prior brain surgery, radiotherapy or chemotherapy. (6) Imaging examinations indicating the presence of brain metastases in patients, with no surgical contraindications. (7) Patients and their families supported this study and signed informed consent forms. (8) For patients with a preoperative Karnofsky Performance Status (KPS) below 70, surgery was reserved for those presenting with either life-threatening mass effect (midline shift >5 mm or impending herniation) or rapidly progressive, potentially reversible neurological deterioration (e.g., acute hemiparesis from a dominant frontal lesion).

The exclusion criteria were as follows: (1) patients with poor liver and kidney function who could not tolerate surgery. (2) Patients allergic to fluorescein sodium. (3) Patients who could not undergo magnetic resonance examination and had not made a preoperative navigation plan. All patients in the study completed Preoperative auxiliary examinations which included head computed tomography (CT), plain and enhanced magnetic resonance imaging (MRI), magnetic resonance angiography (MRA), magnetic resonance venography (MRV), magnetic resonance diffusion-weighted imaging (DWI), magnetic resonance diffusion tensor imaging (DTI), and magnetic resonance spectroscopy (MRS). This study was approved by the Medical Ethics Committee of Inner Mongolia Autonomous Region People’s Hospital (202411811L). All patients with brain metastases who participated in the study signed an informed consent form.

### 2.2 Preoperative navigation plan preparation

Comprehensive preoperative imaging examinations were conducted, including multiplanar CT, MRI with enhanced sequences, CTA, DTI, MRA, MRV, and DWI of the head. The imaging data were imported into the Brainlab functional neurological navigation software. After tracing, the precise location of the tumor tissue and its spatial relationship with important nerve fiber conduction tracts, pyramidal tracts, and large blood vessels were determined. Image fusion was performed to establish a surgical navigation plan.

### 2.3 Intraoperative navigation

Based on the preoperative customized navigation plan, real-time localization of the tumor location and the relationship with the pyramidal tract and blood vessels was performed during the operation to minimize damage to normal brain tissue and blood vessels (**Figures 1A–D**).

**Figure 1:**
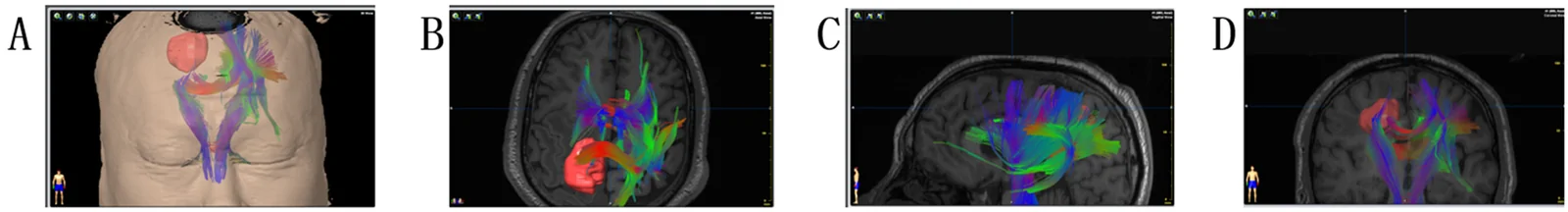
Intraoperative navigation images of brain metastases, showing the relationship between the tumor and the conducting tracts and blood vessels. **(A)** 3D display; **(B)** axial position; **(C)** sagittal position; **(D)** coronal position.

### 2.4 Sodium fluorescein was sourced from Guangxi Wuzhou pharmaceutical factory (3 ml: 0.6 mg)

Following induction of general anesthesia, a standardized allergy test was performed. A 20% sodium fluorescein injection (0.5 mL) was diluted to 5 mL with 0.9% normal saline. Subsequently, 0.5 mL of the diluted solution (equivalent to 0.05 mL of the original 20% solution) was administered intravenously as a test dose. The patient was observed for 10 minutes. A positive hypersensitivity reaction was defined as the occurrence of any of the following in the absence of concurrent anesthetic boluses or surgical stimulation: (i) ≥20% change in mean arterial pressure or heart rate from baseline; (ii) cutaneous flushing, urticaria, or angioedema; (iii) bronchospasm, wheezing, or oxygen desaturation; or (iv) sustained cardiac arrhythmia. Rescue measures (intravenous antihistamines, corticosteroids, or vasopressors as indicated) were prepared and would be implemented immediately upon a positive test. Among the 40 patients in the observation group, none exhibited a positive reaction, and no patient was excluded because of suspected fluorescein hypersensitivity.

For patients with a negative test, sodium fluorescein was administered intravenously at a dosage of 2 mg/kg. The median interval from fluorescein injection to skin incision was 10 minutes (range, 0–20 minutes), to dural opening was 90 minutes (range, 75–110 minutes), and to first microscopic visualization of tumor fluorescence under the Y560 filter was 98 minutes (range, 82–120 minutes). These intervals were recorded prospectively by the anesthesia team. This timing protocol ensures that the dye has adequately distributed into the peritumoral extracellular space by the time the dura is opened, thereby enhancing the clarity of the tumor–brain interface at the moment of microscopic resection.

Intraoperative fluorescence was assessed using a semi-quantitative three-tier grading system under the Y560 filter: Grade 0 (absent or weak fluorescence, barely distinguishable from background), Grade 1 (moderate fluorescence, clearly visible yellow staining), and Grade 2 (intense fluorescence, bright yellow staining with sharp demarcation). This assessment was performed independently by the operating surgeon and the assistant surgeon; discrepancies were resolved by consensus.

During the operation, the microscope was switched to the YELLOW 560 fluorescence mode. Tumor tissue was visible as prominently yellow-stained, with a distinct boundary separating it from normal brain tissue (**Figures 2A, B**). Technical nuances of Y560 filter use included: (i) minimizing ambient operating-room light prior to fluorescence assessment; (ii) brief interruption of white-light illumination to visualize yellow fluorescence against a dark background; (iii) avoidance of simultaneous irrigation with lactated Ringer’s solution, which can dilute surface fluorescence and reduce contrast; and (iv) confirmation of fluorescence boundaries under low magnification before proceeding with high-magnification microdissection. The Y560 long-pass filter module (excitation 460–500 nm, emission >560 nm) was integrated into the OPMI Pentero 900 microscope (Carl Zeiss, Oberkochen, Germany).

**Figure 2:**
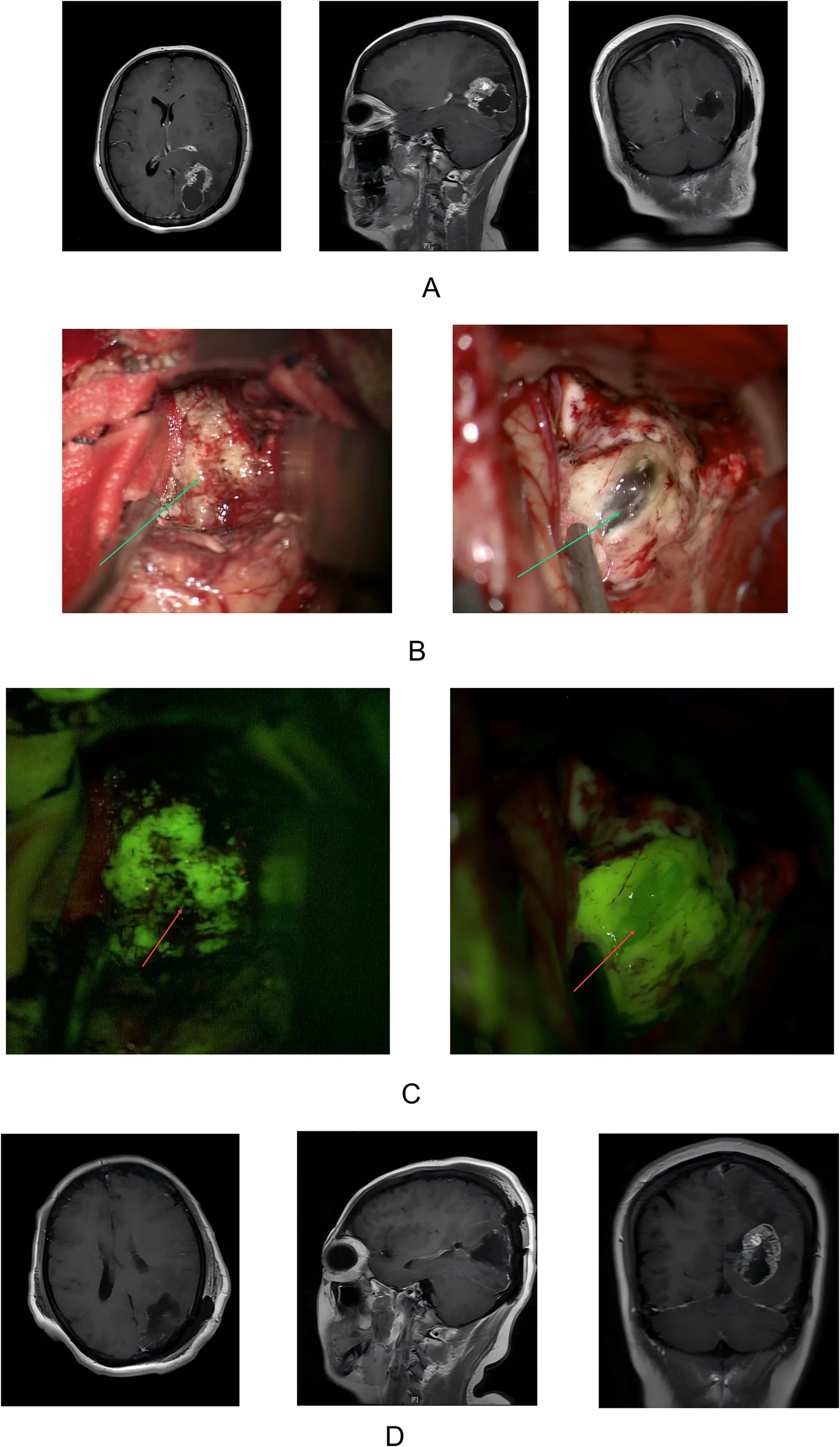
Representative cases illustrating fluorescein-guided resection of brain metastases. For each patient, the panel set shows: **(A)** preoperative contrast-enhanced T1-weighted MRI; **(B)** intraoperative white-light microscopy; **(C)** Y560-filtered fluorescence view; and **(D)** postoperative contrast-enhanced MRI confirming the resection cavity. Scale bars and arrows indicate the fluorescence-tumor interface. The yellow fluorescence demarcates the metastatic tissue from surrounding brain, including regions where white-light visualization alone provided poor contrast.

During resection, a neuronavigation probe was used to explore tumor boundaries. Forceps were used to grasp tumor tissue at the boundary, where yellow staining transitioned from obvious to faint, and samples were sent for intraoperative frozen-section analysis. After estimating complete tumor resection, the microscope was converted to YELLOW 560 fluorescence mode to inspect the tumor cavity. The absence of yellow staining indicated that the resection had reached the tumor boundary. At this point, the tumor boundaries were reassessed using the navigation probe to achieve maximal safe resection. Postoperative tumor tissues were sent for pathological and molecular examinations.

### 2.5 Observation indicators

The degree of tumor resection after surgery, improvement in Karnofsky performance status (KPS) score, postoperative complications, the occurrence of new neurological dysfunctions, operation time, and length of hospital stay were observed (1). All patients underwent head-enhanced MRI 72 hours after surgery. The tumor resection rate was calculated and compared with the preoperative examinations. As shown in **Figures 2C, D**, the enhanced part of the T1-weighted sequence was used as the standard, and the largest plane of the tumor was selected. Tumor volumes before and after surgery were calculated for comparative analysis (7). The formula for calculating the tumor volume (V) was V = A × B × C/2, where A, B, and C are the maximum lengths of the MRI axial, sagittal, and coronal scans, respectively. Postoperative MRI was independently reviewed by two experienced neuroradiologists, blinded to group assignment. Tumor volume was calculated using the formula V = A × B × C/2. In case of discrepancies, a third senior neurosurgeon was consulted to reach consensus (see Section 1.5 of the Methods). Final assessments were confirmed jointly by a neurosurgeon and a neuroradiologist.The evaluation criteria for resection degree were as follows (8): Total resection: The resection rate reaches 100% or an expanded resection is performed to remove the tumor tissue completely. Subtotal resection: The resection rate exceeds 90%, however, does not reach 100%. Partial resection: The resection rate was 60–and 90% (2). Based on the KPS score, we compared improvements in neurological dysfunction in patients before and after surgery (3). Postoperative complications, such as intracranial infection, pulmonary infection, and epilepsy, occur in patients after surgery. Imaging examinations were conducted at 3, 6, and 12 months after surgery to evaluate the tumor condition and resection effect (4). The operation time and length of hospital stay in both groups were observed (5). All patients in the group were followed up at 3, 6, and 12 months postoperatively, during which head-enhanced MRI was re-examined, and the mortality rates of the two groups were counted.

### 2.6 Statistical methods

Statistical analysis of the data was carried out using Statistical Package for the Social Science software version 26.0. The classified data were analyzed using chi-square test, reporting rate and 95% confidence interval (normal approximation method). Continuous data that follow a normal distribution were tested by t-test, and the mean ± standard deviation and 95% confidence interval were reported. One-way analysis of variance was used to compare the means among different groups. Statistical significance was set at P< 0.05.

## 3 Results

There were no statistically significant differences between the two groups in terms of sex (male/female), age (years), preoperative KPS score, location of intracranial tumors (supratentorial or infratentorial), number of intracranial metastases, presence or absence of extracranial metastases, pathological type, operation time, or length of hospital stay (P > 0.05), suggesting that the data are comparable (**Table 1**).

In the observation group, all 40 patients tolerated the fluorescein allergy test without evidence of hypersensitivity; consequently, no patient was excluded from the study because of a positive test. Intraoperative fluorescence was visible in all resected lesions. According to the semi-quantitative grading system, intense fluorescence (Grade 2) was observed in the majority of cases, whereas moderate fluorescence (Grade 1) was noted in a small subset of lesions, typically those with cystic degeneration or deep subcortical location. No lesion exhibited absent fluorescence. All preoperatively contrast-enhancing metastases demonstrated visible intraoperative fluorescence under the Y560 filter.

In the observation group, none of the patients exhibited an allergy to sodium fluorescein. This group had a total of 14 complications. Three patients presented with clinical symptoms, such as high fever and consciousness disorders, after surgery. A lumbar puncture indicated an intracranial infection and after receiving anti-infective treatment, their symptoms improved. Nine patients had pulmonary infections and two had epilepsy, all of whom had tumors located close to the motor area. In the control group, 18 patients experienced postoperative complications such as epilepsy, pulmonary infection, and intracranial infection. After treatment, symptoms improved in all cases. There were no statistically significant differences in the complications between the two groups.

All patients underwent preoperatively navigation plans to determine the positional relationship between the tumor tissue, important blood vessels, and neural conduction tracts. During resection, the positional relationship between the tumor, blood vessels, and conduction bundles was verified. Satisfactory results were obtained in the surgery for resecting brain metastases through the combined application of neuronavigation and fluorescein sodium in the observation group. After conducting a head-enhanced MRI 3 days postoperatively and comparing it with the preoperative imaging information, the tumor resection rate was calculated. The resection rate in the observation group was significantly higher than that in the control group (90% [95% CI: 80.6%, 99.4%] vs. 55% [39.4%, 70.6%]), and this difference was statistically significant (*X*^2^ = 6.144, P = 0.013). The improvement in the postoperative KPS score in the observation group was greater than that in the control group (85%[95% CI: 70.9%, 93.0%] vs. 55%[95% CI: 40.0%, 69.9%]), and the difference was statistically significant (*X*^2^ = 4.286, P = 0.038). The incidence of new postoperative functional disorders was significantly lower in the observation group than in the control group (10%[95% CI: 4.1%, 23.0%] vs. 40%[95% CI: 24.8%, 55.2%]) (*X*^2^ = 4.800, P = 0.028). Subgroup analysis by primary tumor histology indicated that visible intraoperative fluorescence was present in all histotypes (lung adenocarcinoma, gastrointestinal, genitourinary, breast). While the proportion of intense fluorescence varied slightly among groups, these differences were not statistically significant (P > 0.05), and all histotypes achieved comparable gross-total resection rates under combined guidance.

Among the 40 patients in the fluorescein group, 8 had tumors adjacent to eloquent cortex (e.g., motor or language areas), compared to 7 in the control group. Postoperative functional deficits were observed in only 1 of 8 patients (12.5%) in the fluorescein group, compared to 3 of 7 (42.9%) in the control group, highlighting the potential role of fluorescein guidance in preserving neurological function in eloquent areas. Although the number of postoperative complications was lower in the observation group than in the control group (35%[95% CI: 22.2%, 50.0%] vs. 45%[95% CI: 31.0%, 60.4%]), the difference was not statistically significant (*X*^2^ = 0.833, P = 0.361). The operation time in the observation group (216.65 ± 56.76 min95% CI:199.03, 234.27) was shorter than that in the control group (225.62 ± 37.68 min95% CI:217.89, 233.35), yet this difference was not statistically significant (t = 0.589, P = 0.560). The hospital stay in the observation group (21.48 ± 3.23 days95% CI:20.49, 22.47) was shorter than that in the control group (22.43 ± 2.34 days95% CI:21.71, 23.16), however, the difference was not statistically significant (t = 1.065, P = 0.294). More detailed information is provided in **Table 2**. No surgical-related mortality occurred in either group. Both groups of patients were followed up for 1 year, and the1-year survival rates were compared. The 1-year survival rate in the observation was significantly higher than in the control groups (67.5% 27/40, 95% CI: 52.4%, 82.6%vs. 42.5% 17/40, 27.1%, 57.9%). More detailed information is provided in **Table 2**. Detailed data on postoperative adjuvant therapies—including types of radiotherapy (WBRT, SRT, SRS) in **Table 3**.Future studies should be carried out to evaluate the long-term follow-up influence of surgical methods on the survival and recurrence rates of both groups of patients.

**Table 2:** Comparison of surgical data between the observation group and the control group.

Clinical characteristics	Control group (n=40)	Observation group (n=40)	Statistical value	P value
Degree of tumor resection	90.0%	55.0%	χ^2^ = 6.144	0.013
Total resection	22	36		
Subtotal resection	18	4		
Improvement of KPS score			χ^2^ = 4.286	0.038
Improved	22	34		
Unchanged	18	6		
Postoperative complications			χ^2^ = 0.833	0.361
Present	18	14		
Absent	22	26		
New-onset functional impairment	10.0%	40.0%	χ^2^ = 4.800	0.029
Present	16	4		
Absent	24	36		
Surgical time ( $\bar{x}\pm \text{s}$, min)	225.62 ± 37.68	216.65 ± 56.76	t=0.589	0.560
Length of hospital stay ( $\bar{x}\pm \text{s}$, d)	22.43 ± 2.34	21.48 ± 3.23	t=1.065	0.294
One-year survival rate	17	27	5.051	0.025

**Table 3:** Postoperative adjuvant therapies in the two groups.

Clinical characteristics	Control group (n=40)	Observation group (n=40)	Statistical value	P value
Number of patients receiving radiotherapy (cases)	22	28	χ^2^ = 1.234	0.266
SRS (cases)	15	20		
WBRT(cases)	7	8		
Chemotherapy (cases)	10	12	χ^2^ = 0.321	0.571

## 4 Discussion

With advances in the diagnosis and treatment of systemic cancers, patient survival has improved substantially, and the incidence of brain metastases has increased correspondingly (2). Although targeted therapy, immunotherapy, and stereotactic radiosurgery have expanded the therapeutic options for these patients (8), surgical resection retains a central role because it rapidly relieves mass effect, improves quality of life, and provides tissue for histopathological diagnosis (3, 9, 10).

Accepted indications for craniotomy include large metastases (>3 cm in diameter); impending herniation from severe peritumoral edema or intratumoral hemorrhage; lesions suspected to be metastases without a confirmed primary pathology; and cystic metastases refractory to radiotherapy, chemotherapy, or targeted therapy (11–13). In patients with severe headache, impaired consciousness, hemiplegia, or other signs of elevated intracranial pressure, surgery promptly relieves intracranial hypertension and reverses neurological deficits. Moreover, molecular characterization of the specimen guides subsequent targeted therapy and immunotherapy. Conversely, small, deep-seated, asymptomatic metastases in non-eloquent regions are generally managed with stereotactic radiosurgery (SRS) or whole-brain radiotherapy (WBRT) (8, 9).

Accurate delineation of the tumor margin is a prerequisite for maximizing safe resection while preserving neurological function (14–16). Multimodal functional neuronavigation integrates the actual tumor position with multiple preoperative image datasets, providing real-time guidance to protect eloquent structures such as the pyramidal tract and major vessels, and has been widely adopted in neurosurgical practice (17). Neuronavigation is nevertheless limited by brain shift, which results from cerebrospinal fluid egress, reduced intracranial pressure, and surgical traction and progressively degrades registration accuracy. To mitigate this effect, we minimized the intraoperative use of osmotic diuretics such as mannitol to preserve cerebrospinal fluid volume, and combined navigation with real-time fluorescein guidance and intraoperative ultrasound to compensate for residual shift and improve the extent of resection.

Fluorescence-guided surgery is another widely used adjunct for tumor resection. The agents most commonly used clinically are 5-aminolevulinic acid (5-ALA), sodium fluorescein (SF), and indocyanine green (ICG) (18, 19). Unlike 5-ALA, which accumulates within tumor cells, SF is a nonspecific fluorophore distributed in the extracellular compartment wherever the blood-brain barrier (BBB) is disrupted (20); when the BBB is intact, the dye does not extravasate, whereas barrier disruption in tumor tissue permits its accumulation and clear demarcation of the lesion. Since Moore et al. first reported fluorescein imaging of intracranial lesions in 1948, the relevant literature has expanded to encompass gliomas, brain metastases, pituitary tumors, lymphomas, and meningiomas (14, 16, 21, 22). As Musca et al. demonstrated, fluorescein accumulation in tumor tissue is not merely a marker of BBB disruption: its intracellular detection suggests selective uptake by tumor cells, and the mechanisms underlying its differential uptake among cell types of the tumor microenvironment warrant in-depth investigation (23).

Comparison of SF and 5-ALA in Brain Metastases Surgery. While 5-ALA offers high specificity for neoplastic tissue and has been supported by previous studies in glioblastoma surgery, several practical limitations restrict its universal adoption: it requires specialized blue-light filter systems, strict control of ambient operating-room illumination, is associated with higher direct costs, and carries a risk of phototoxicity. Sodium fluorescein, by contrast, is inexpensive, widely accessible, compatible with standard Y560 long-pass filters on conventional operating microscopes (e.g., Zeiss Pentero series), and requires no modification of operating-room lighting. It enables real-time delineation of tumor boundaries under standard fluorescence microscopy without additional capital equipment. We therefore posit that SF represents a pragmatic, cost-effective alternative for centers where 5-ALA is not routinely available, and that the two agents may serve complementary roles depending on institutional resources and tumor histology.

Compared with conventional white-light microscopy, SF fluorescence enhances tumor visualization, increases the extent of resection, reduces recurrence, and improves postoperative quality of life and survival. Studies by Kofoed, Kerschbaumer, Ferreira, Cheng, and others have likewise confirmed the value of fluorescein-guided resection in improving tumor visualization and surgical outcomes (15, 16, 24, 25). Whereas most prior studies examined fluorescence or neuronavigation in isolation, the present study evaluates the integration of low-dose SF (2 mg/kg) with multimodal functional neuronavigation for brain metastasis surgery. Because prior fluorescein studies focused largely on gliomas (6), in which the mechanisms of BBB disruption differ from those in metastatic lesions, the fluorescence behavior of brain metastases merits separate analysis. A comparative study reported a higher complete resection rate with SF than with 5-ALA (78% vs 65%), consistent with the rates observed in our cohort (90% vs 55%) (15).

Recent studies have administered low-dose SF (1–2 mg/kg) (29, 30), although the recommended dose in most published series remains 5 mg/kg (31, 32). The 2 mg/kg dose selected in this study was based on emerging evidence that lower doses provide sufficient intraoperative visualization while minimizing systemic adverse effects. This dosing strategy was further reinforced by our institutional experience; the allergy test protocol was adopted as a safety measure following an internal review of a prior case of transient anaphylaxis associated with standard-dose (5 mg/kg) fluorescein administration. The reduced dose, combined with a rigorous pre-administration test, aligns with contemporary trends toward safer, lower-dose fluorescence protocols (29, 30). In our experience, intravenous administration before skin incision achieves optimal visualization during resection.

Intraoperatively, regions of preoperative T1-weighted contrast enhancement displayed yellow staining. Some FLAIR-hyperintense areas also exhibited fluorescein staining. While this observation raises the intriguing possibility of peritumoral tumor infiltration or reactive BBB disruption, we caution that this interpretation is hypothesis-generating only. No histopathological confirmation of infiltrated tissue was obtained in this study, and FLAIR hyperintensity may reflect vasogenic edema rather than neoplastic spread. This limitation should be considered when interpreting the biological significance of FLAIR-positive fluorescence. Residual fluorescence at the cavity margin prompted further resection, whereas staining of the ependyma and dura mater was regarded as physiological.

In the present study, the observation group achieved a significantly greater extent of resection than the control group, with higher KPS scores at 3 months and fewer new neurological deficits, whereas complications did not differ between groups, indicating that SF guidance adds no procedural risk. No SF-related adverse events occurred, and postoperative MRI confirmed the improved resection rate; together, these findings support the safety and feasibility of SF-guided resection of brain metastases. Compared with 5-ALA and ICG, SF offers practical advantages, including low cost, lack of toxicity, simple administration, and broad applicability across central nervous system tumors, and enables real-time delineation of the tumor boundary, allowing surgeons to mark the lesion and improve resection (25, 26). Falco et al. showed that 2 mg/kg of SF enables visualization of infiltrative micronodules<1 mm in diameter under fluorescence microscopy, consistent with the high positivity rates of intraoperative frozen sections from fluorescein-bright margins in our series (27). A multicenter study by Schebesch et al. further demonstrated that SF guidance reduces residual tumor rates by 40%, providing a mechanistic basis for the increased resection rate we observed (28, 29).

Multimodal functional neuronavigation enables accurate preoperative planning and intraoperative localization, clarifying the spatial relationships among the tumor, the surrounding brain parenchyma, blood vessels, and white-matter tracts. Resection under yellow-fluorescence microscopy, in turn, compensates for the localization error introduced by brain shift after cerebrospinal fluid loss and delineates the true tumor boundaries in real time. We therefore consider the combination of these two techniques superior to conventional microscopic resection under direct vision: it enables more accurate localization and more complete removal of brain metastases while safeguarding neurological function, thereby reducing postoperative neurological deficits and improving prognosis without increasing procedure-related complications (30, 31).

This study has several limitations that should be acknowledged. First, the retrospective, single-center, historical cohort design introduces inherent selection bias. Improvements in surgical experience, perioperative management, and adjuvant oncological therapies over the 6-year enrollment period may have contributed to the observed outcomes, despite propensity score matching. Second, tumor volumes were estimated with the ellipsoid formula, which is less accurate than three-dimensional volumetric analysis; dedicated MRI quantification software will be used in future studies. Third, although most lesions were located in non-eloquent regions, a few bordered functional areas, and intraoperative neurophysiological monitoring (e.g., cortical stimulation, motor evoked potentials) was unavailable at our center owing to technical constraints; its absence may have limited functional protection in these cases. Fourth, the assessment of fluorescence intensity, although standardized by our three-tier grading system, retains a subjective component. Fifth, the observation of FLAIR-positive fluorescence was not validated histopathologically and should be interpreted with caution. Finally, the sample size was modest and no *a priori* power calculation was performed, limiting statistical robustness and generalizability. A multicenter randomized controlled trial with extended follow-up is warranted to validate these findings.

## 5 Conclusion

Multimodal functional neuronavigation combined with intraoperative low-dose sodium fluorescein guidance is a safe and feasible strategy for the resection of brain metastases and, in this study, was associated with better surgical outcomes than conventional microscopic resection. Sodium fluorescein enhanced intraoperative tumor visualization and increased the extent of resection, while neuronavigation clarified the spatial relationships among the tumor, adjacent vasculature, white-matter tracts, and eloquent cortex, facilitating maximal resection with preservation of neurological function. Accordingly, patients treated with the combined approach achieved a greater extent of resection, better early postoperative functional status, and fewer new neurological deficits, without an increase in complications (32). Nevertheless, given the retrospective historical cohort design, the non-uniform distribution of surgical teams between groups, and the potential confounding effects of primary tumor biology, molecular status, and postoperative adjuvant therapies, these findings should be interpreted with caution and warrant confirmation in prospective, randomized studies.

## Data Availability

The original contributions presented in the study are included in the article/supplementary material. Further inquiries can be directed to the corresponding author.
